# Oral Realgar-Indigo Naturalis Formula Plus Retinoic Acid for Acute Promyelocytic Leukemia

**DOI:** 10.3389/fonc.2020.597601

**Published:** 2021-02-05

**Authors:** Yinjun Lou, Yafang Ma, Jie Jin, Honghu Zhu

**Affiliations:** Department of Hematology, Leukemia Center, The First Affiliated Hospital of Zhejiang University, College of Medicine, Key Laboratory of Hematopoietic Malignancies in Zhejiang Province, Hangzhou, China

**Keywords:** realgar-indigo naturalis formula, all-trans retinoic acid, PML-RARA, arsenic trioxide, chemotherapy-free

## Abstract

Treatment paradigm of acute promyelocytic leukemia (APL) is by no mean the most remarkable story of cancer therapy. Recently, the advent of oral arsenic formulations (oral-arsenic trioxide and Realgar-Indigo Naturalis formula (RIF)) based regimens may provide a therapeutic advance by curing APL with two oral agents. Indeed, the oral RIF plus all-trans-retinoic acid (ATRA) without chemotherapy display highly efficacy in patients with APL. The safety profile of RIF plus ATRA make possible to treat APL patients in a home-based manner during postremission therapy. To our knowledge, RIF was the first commercially available oral arsenic agent approved in China. The RIF plus ATRA regimens are becoming a preferred frontline care for APL in China. In this review, we will discuss the history, current evidences and challengers of RIF-based strategies in APL. More and more APL patients may enjoy a cure with a normal quality-of-life after induction in the near future.

## Introduction

Acute promyelocytic leukemia (APL) was first described  in 1923 by Swiss hematologist Dr. Albert Alder (1). In the 1950s, LK Hillestad and J Bernard further described a case series and recognized as a clinical entity of APL by its unique morphologic characteristics with prominent granules and hemorrhagic diathesis features (2, 3). Importantly, the initial breakthrough was the uncover of the unique chromosomal aberration t(15, 17) (q22;q12) in APL by Dr. J Rowley in 1970s (4). However, it was not until 1990, the landmark molecular fusion of the promyelocytic leukemia (PML) and retinoic acid receptor alpha (RARA) gene was identified (5). Given the central role of PML-RARA in the leukemogenesis of APL, the chimeric oncoprotein was considered an idea molecular target for antileukemic therapy.

Surprising, the targeted therapy to PML-RARA for APL was developed by an empirical-based way. As early as in 1985, all-trans-retinoic acid (ATRA) was first administrated in the induction treatment of APL by Dr. Wang ZY and his team from Shanghai Institute of Hematology (6). The combination of ATRA and anthracycline-based protocol significantly improved the long-term outcome of APL (7). In 1970s, another miracle agent, intravenous arsenic trioxide (ATO) was observed preliminary evidence of efficacy in APL patients by Dr. Zhang TD et al. from Harbin Medical University (8). Subsequent studies by Dr. Chen Z et al. confirmed the high efficacy and safety of intravenous ATO for APL, initially in relapsed patients and then for newly diagnosed patients (9–11).

Along with the clinical progress, basic investigators have revealed that both ATO and ATRA directly targeted the PML-RARA, which trigger the degradation of the PML-RARA fusion oncoprotein (12). Additionally promising, ATO was found to target PML protein directly (13). The targeted therapy model assumed to eradicate the APL-initiating cells and ultimately reduce the need for chemotherapy (14). In line with this preclinical model, the follow-up trials demonstrated that the ATO plus ATRA targeted approach could definitively cure APL (14). Thus, ATRA and arsenic were defined as a “magic bullet” for APL.

## Oral Realgar-Indigo Naturalis Formula Development and Pilot Studies

Realgar (contains approximately 90% As_4_S_4_), an oral form of arsenic compound, is an agent originally used as traditional Chinese medicine. As early as in 1980s, Dr. Huang SL et al. initially developed a Realgar-Indigo Naturalis Formula (RIF) for APL. RIF (250 mg per pill) was the combination of four natural products: Realgar (30 mg per pill), Indigo naturalis (125 mg per pill), Radix salviae miltiorrhizae (50mg per pill) and Radix pseudostellariae (45 mg per pill).

Similar to ATO, As_4_S_4_ induces the ubiquitination and degradation of PML-RARA, and relocalization of PML protein in primary APL cells was observed. Interestingly, the four components of RIF could make synergistic effect and strengthen the antileukemia effect (15). Clinical data accumulated over the last decades of RIF-based therapy in APL. Here, **Table 1** summarized the main publication data of RIF-based treatment for APL.

**Table 1:** Summary of the main publications of Realgar-Indigo Naturalis Formula (RIF)-based regimens in the treatment of acute promyelocytic leukemia (APL).

Study	Trials	Period	No. of patients	High-risk	Age, median (range)	Induction	Postremission	Chem-free	CR (%)	Median follow-up (m)	Long-term outcomes
**Huang et al. (** 16 **)**	Retro	1988–1993	60 (R/R=17)	NA	31(5-72)	RIF	Chem	No	98.3	NA	NA
**Lu et al. (** 17 **)**	Retro	1994–2000	129 (CR1=123; R/R=7)	NA	NA	As4S4	As_4_S_4_	Yes	100	23	6-year DFS= 87.4%
**Lin et al. (** 18 **)**	Pros; phase 2	2002–2004	61	NA	35(23–47)	RIF	NA	No	96.7	NA	NA
**Zhu et al. (** 19 **)**	Pros; phase 3	2007–2011	114	21	33(15–60)	RIF+ATRA	RIF/ATRA/Chem	No	99.1	61	7-year EFS=93.7%; 7-year OS=95.4%
**Yang MH et al. (** 20 **)**	Pros; phase 3	2011–2017	40	8	9.9 (2–16)	RIF+ATRA	RIF/ATRA/Chem	No	100	36	5-year EFS = 100%; 5-year OS =100%
**Zhu et al. (** 21 **)**	Pros; phase 2	2013–2014	20	0	35(20–58)	RIF+ATRA	RIF/ATRA	Yes	100	14	NA
**Zhu et al. (** 22 **)**	Pros; phase 3	2014–2015	69	0	34 (24–47)	RIF+ATRA	RIF/ATRA	Yes	100	32	2-year EFS= 97%
**Zhu et al. (** 23 **)**	Pros; phase 2	2014–2016	20	20	36(16–61)	RIF+ATRA	RIF/ATRA	Yes	100	33	3-year OS =100%; 3-year EFS 89.4%
**Lou et al. (** 24 **)**	Retro	2015–2019	142	28	41(14–81)	ATO/RIF+ ATRA	RIF/ATRA	Yes	NA	20.7	2-year DFS =99%

Chem, Chemotherapy; CR, complete remission; DFS, disease-free survival; EFS, event-free survival; m, month; NA, not available; No., number; OS, overall survival; Pros, Prospective; Retro, Retrospective; RFS, relapse-free survival; RIF, Realgar-Indigo Naturalis Formula.

In 1995, Dr. Huang SL et al. undertook a pilot study of APL patients treated by oral RIF based induction in six Chinese centers from 1988 to 1993 (16). Forty-three patients were newly diagnosed APL and 17 were relapsed APL. Patients received RIF till completely remission (CR) or 60 days. The dose was started with 15 pills daily and gradually ramp-up to 30 pills daily in a week. Glucocorticoid or low-dose chemotherapy was allowed. The overall CR rate was 98.3%. All patients have received RIF more than 30 days achieved CR. Moreover, the side effects of RIF appeared generally manageable. The frequent main side effects were epigastria discomfort, abdominal distention, mild diarrhea, skin rash, and liver enzyme increase. This is the first study reported the efficacy and safety of RIF-based therapy in APL. However, the postremission therapy was not clearly defined and long-term outcomes were not reported.

Subsequently, a team led by Dr. Lu DP from Peking University, first isolated highly purified crystalline As_4_S_4_ from natural Realgar. A total of 129 APL patients were enrolled during 1994–2000 (17). During induction, As_4_S_4_ was orally administered at a dosage of 50 mg/kg/day (four times daily) until CR. After CR1, patients received As_4_S_4_ single agent for postremission therapy. The results were quite encouraging. In the newly diagnosed group (n=15), the 3-year disease-free survival (DFS) rates was 76.6%. In the CR1 group (n=99), the 6-year DFS rates were 87.4%. The data suggested that oral As_4_S_4_ single agent could cure more than 70% of APL patients. This seems consistent with APL patients using single intravenous ATO regimen.

In terms of toxicity, As_4_S_4_ was usually well tolerated, such as asymptomatic QTc prolongation (33%), transient elevation in liver enzyme levels (10.5%), mild nausea (3.2%), skin itching (3.2%). No myelosuppression occurred during postremission therapy. Pharmacokinetic and pharmacodynamic analysis of As_4_S_4_ was performed in seven patients. Both blood and urinary arsenic levels eliminated after discontinuation of As_4_S_4_.

## Randomized Trials: Oral Realgar-Indigo Naturalis Formula Versus All-Trans-Retinoic Acid

To further evaluate the effective and side effects of oral RIF in APL, the Chinese APL Cooperative Group conducted a prospective Phase II trial to compare the efficacy and safety of RIF versus ATRA based induction therapy in patients with newly diagnosed APL (18). During induction, patients received RIF (dose ramp up from 15 pills/day to 30 pills/day in 10 days), or ATRA at 25 mg/m^2^/day (in two divided doses). Low intensive chemotherapy was used for cytoreduction. By intention-to- treat (ITT) analysis, the CR rate was 80.8% in RIF group versus 75.7% in the ATRA group (P > 0.05). The trial suggested that RIF was an alternative choice of APL. The main limitation of the study was not available of long-term outcomes.

## Randomized Trials: Oral Realgar-Indigo Naturalis Formula Plus All-Trans-Retinoic Acid Versus Intravenous Arsenic Trioxide Plus All-Trans-Retinoic Acid

Based on the phase II trial, the Chinese APL Cooperative Group further conducted a phase III multicenter, randomized, prospective study between 2007 and 2011 (ChiCTR-TRC-12002151), which compared RIF plus ATRA versus ATO plus ATRA based regiment in front-line therapy of all-risk APL (19). During induction, patients were given oral RIF (60 mg/kg/day, orally in three divided doses) or ATO (0.16 mg/kg) combined with ATRA (25 mg/m^2^/day, orally in two divided doses). On achieving CR1, patients received three cycles of chemotherapy and maintenance with sequential RIF or ATO plus ATRA for 2 years. The study was a noninferiority design and the primary end point was DFS.

A total of 242 patients were enrolled at seven centers. The 2-year DFS was 98.1% in the RIF group versus 95.5% in the ATO group and confirmed the noninferiority (P <0.001). Similar hematologic and non-hematologic adverse events were reported. Moreover, pharmacodynamics analysis showed oral RIF were similar to intravenous ATO. Thus, this is the first study demonstrate that an oral RIF and intravenous ATO have broadly similar efficacy and safety. In addition, the updated results revealed the estimated 7-year EFS and OS rates were similar between the RIF and ATO groups (94% versus 89%, P = 0.37; 95% versus. 91%, P = 0.31, respectively) (25). The estimated 7-year EFS and OS were also similar between the high-risk and non-high-risk groups (25).

Since the oral RIF plus ATRA may be easier for pediatric patients to take, the South China Children Leukemia Group (SCCLG) conducted a randomized study to compare the efficacy, safety and the number of hospital days between RIF or f intravenous ATO-based therapies in pediatric APL (20). The induction and consolidation treatment contained ATO or RIF plus ATRA and low intensity chemotherapy. The RIF was given at a dose of 135 mg/kg/day (≯30 pills/day). Intravenously ATO was given at a dose of 0.16 mg/kg/day (≯10 mg/day). The results showed the 5-year EFS was 100% in both groups. The data suggested that oral RIF was similar efficacy and safe with intravenous ATO in pediatric APL patients, with the advantage of reducing hospital stay days.

Base on the phase II trial and the phase III trial, the therapeutic effect of RIF in APL is well established. The Chinese Food and Drug Administration approved RIF as the first-line treatment for APL. RIF was implemented into the treatment algorithm and widely used for APL in China. The optimal recommended dose of RIF was 60 mg/kg/day in adult.

## Chemotherapy-Free Approach: Oral Realgar-Indigo Naturalis Formula Plus All-Trans-Retinoic Acid

Although the Chinese APL07 trial achieved excellent long-term outcomes in newly diagnosed APL, the three cycles of consolidation chemotherapy have been associated with myelosuppresion, risk of cardiovascular complications and the development of therapy-related myeloid neoplasms. In 2002, Estey and coworkers from M.D. Anderson Cancer Center published the results of the combined ATRA with intravenous ATO regimen without chemotherapy regimen (only plus gemtuzumab ozogamycin for high risk patients) in newly diagnosed APL (26). The trial suggested that APL patients could be cured by targeted therapy alone. In 2013, the landmark APL0406 trial demonstrated the superior efficacy, safety and quality of life for ATO-ATRA in comparison with the ATRA plus chemotherapy regimen in non-high risk APL patients (27, 28). Thus, the chemotherapy-free approach was moving to the frontline in APL treatment.

Based on the previous intravenous ATO and RIF data, it is logical to apply oral RIF plus ATRA chemotherapy-free in newly diagnosed APL patients. Zhu HH et al. conducted a single arm pilot study of using the oral RIF plus ATRA protocol in non-high risk patients (26). The trial enrolled 20 patients. Oral RIF was administered (60 mg/kg/day) and ATRA (25 mg/m^2^/day) as induction therapy. Postremission therapy schedule was RIF with a 4 weeks on and 4 weeks off and ATRA 2 weeks on and 2 weeks off for 7 months. All patients achieved molecular CR. No patients relapsed during the cut-off date of last follow-up (20).

Subsequently, a multicenter, non-inferiority, randomized, controlled phase 3 trial were conducted at 14 centers in China (22). The trial compared directly of oral RIF plus ATRA with intravenous ATO plus ATRA in newly diagnosed non-high-risk APL patients. The primary outcome was EFS at 2 years. A total of 109 patients were assigned to RIF-ATRA group (n=72) or intravenous ATO-ATRA group (n=37). The 2-year EFS was 97% versus 94%. The non-inferiority outcome was also confirmed.

## Chemotherapy-Free Approach in High-Risk Acute Promyelocytic Leukemia

Moreover, based on the excellent outcomes of chemotherapy-free approaches in non-high risk APL patients, this raises the question as to whether is it possible for de-escalation or even elimination of chemotherapy for the treatment of high-risk APL patients. Indeed, Zhu et al. conducted an earlier pilot single arm trial with RIF plus ATRA in 20 high-risk patients (23). Minimal cytotoxic agents were allowed during induction. The consolidation approach was RIF (60 mg/kg daily) in a 4-week on and 4-week off regimen for four cycles and ATRA (25 mg/m^2^ daily) in a 2-week on and 2-week off regimen for seven cycles, which was the same postremission schedule as in non-high risk group. The data demonstrated that the 3-year estimated OS and EFS are 100% and 89.4%. Although longer-term follow-up, and more patients needed, this study favored the option of chemotherapy-free regimens in high-risk APL patients.

## Home-Based Management of Patients During Postremission Therapy

Oral RIF and ATRA allow home treatment for APL patients during postremission therapy. The regimen may reduce the cost, hospital/clinic visit and health care resources. It is more convenient for both patients and medical staff, especially in the season while COVID-19 became epidemic. In our experience, the safety profile of oral RIF plus ATRA was favorable during postremission therapy. The side effects are usually mild, such as associated with grade 1/2 neutropenia, gastrointestinal toxicity, and edema. Patients usually come in every 2–4 weeks for blood tests and every three months for bone marrow aspiration (24, 29). Majority of patients could go back to work or school with maintaining normal lives.

## Critical Issues in The Current Chemotherapy-Free Era

However, despite the promising activity of oral arsenic plus ATRA chemotherapy-free approach, several challenges remain. First, early death before or during induction remain the most critical challenge in APL. Early diagnosis, intensive supportive therapy and risk-adapted approach may reduce the early complications during induction. For high-risk patients, anthracyclines, or gemtuzumab ozogamycin may still be necessary to control hyperleukocytosis.

Second, although the main ingredient of RIF is As_4_S_4_, RIF is a compound preparation. Usually, patients need to take about 12–15 pills every day. It would be easy to take if RIF pills were modified by improving producing technology.

Our goal is to cure APL with treatments that are broadly available. RIF is not accessible outside China yet. Fortunately, trials of other oral arsenic formulations are ongoing. Kwong et al. from University of Hong Kong initially use oral-ATO in first relapse, and then in frontline of APL treatment (30). Oral-ATO may be rational to replace the intravenous ATO with emerging evidence increased. Such as, Ravandi et al. conducted a phase I clinical trials of oral-ATO (ORH-2014, NCT03048344) in patients with advanced hematologic malignancies. The Australasian Group also conducted a phase I trial (ACTRN12616001022459) using oral-ATO in APL patients.

Finally, data of chemotherapy-free approach in high-risk patients are still limited. Near future, well-designed clinical trials may possible to optimize the dose and schedule strategy of RIF plus ATRA protocol. The ongoing trials (APOLLO, NCT02688140 by the European intergroup; NCT03624270 by the University of Hong Kong, ChiCTR1900023309 in China) are evaluating the chemotherapy-free approaches in high-risk patients. The results of these trials are very expected indeed.

## Conclusions and Prospective

More than 30 years of experience with RIF, and 10 years since the approval of RIF by the Chinese FDA, the efficacy and safety of RIF-based regiments has been well established (31). Till now, over 8,000 patients with APL have been treated with oral RIF in China. Overall, oral arsenic, a gift from nature, may representative the first oral arsenical formula applied in APL. Near one century after first describe of APL, we believe the treatment of APL is moving toward an entirely oral chemotherapy-free approach among most patients. The oral arsenic plus ATRA will ultimately make treatment safer, less financial burden, and more accessible to patients.

## Author Contributions

LJ, MF, JJ, and ZH conceptualized the study, wrote the manuscript, and gave the final approval of manuscript. All authors contributed to the article and approved the submitted version.

## Funding

This work was supported by grants from the National Natural Science Foundation of China (82070151).

## Conflict of Interest

The authors declare that the research was conducted in the absence of any commercial or financial relationships that could be construed as a potential conflict of interest.
